# Clinical and radiological results comparison of allograft and polyetheretherketone cage for one to two-level anterior cervical discectomy and fusion: A CONSORT-compliant article

**DOI:** 10.1097/MD.0000000000017935

**Published:** 2019-11-11

**Authors:** Sen Yang, Yang Yu, Xun Liu, Zehua Zhang, TianYong Hou, Jianzhong Xu, Wenjie Wu, Fei Luo

**Affiliations:** a Department of Orthopaedics, Southwest Hospital, Third Military Medical University, Chongqing; b Department of Orthopaedics, Hospital of Chengdu University of Traditional Chinese Medicine, Chengdu, Sichuan Province, China.

**Keywords:** allograft, anterior approach, cage, fusion, polyetheretherketone

## Abstract

**Background:**

Recently, many kinds of cages for cervical fusion have been developed to avoid the related complications caused by tricortical iliac crest graft. The existing literature has reported the excellent clinical efficacy and superior fusion rate. However, various types of cages have their own disadvantages. Which bone graft material is the best choice for cage with the fewest complications? At present, there is still no conclusion.

**Methods:**

By reviewing patients with 1 to 2-level cervical degenerative disease in our hospital with a novel cage made of allograft or polyetheretherketone (PEEK), we evaluated the efficacy and reliability of the new cage in anterior cervical discectomy and fusion (ACDF). From 2015 to 2016, a prospective review of 58 and 49 consecutive cases with spondylotic radiculopathy or myelopathy undergoing ACDF using allograft (group A) and PEEK (group B) cage were performed. The follow-up ranged from 12 to 40 months. Intraoperative index, clinical outcome and complications were recorded. Radiographs evaluated segmental and overall cervical lordosis, the height of the intervertebral space, interbody height ratio (IHR), cage positioning, and fusion state.

**Results:**

A total of 134 cages were implanted. Compared to preoperatively, the visual analog scale (VAS) and neck disability index (NDI) were reduced postoperatively without any change during the subsequent follow-up in both groups. There was no migration or extrusion of the cages at the latest follow-up. There were 2 and 4 patients suffering dysphagia respectively. In both groups, the intervertebral height, IHR, segmental and overall cervical lordosis were significantly greater than pre-operation (*P* < .05) and were maintained at the last follow-up, but were not statistically significant (*P* > .05). The allograft group achieved a fusion rate of 100% (58/58) according to CT scans at 3 months post-operation, while PEEK group was 91.8% (45/49), which reached 95.9% (47/49) at 6 months and 100% at 12 months. In addition, the fusion state was maintained in all patients at the last follow-up.

**Conclusion:**

Our data showed that the new allograft cage is superior to the PEEK cage in providing a high fusion rate and fewer complications after 1-level and 2-level ACDF procedures. It may represent an excellent alternative to other cages.

**Highlight**

- The new cage can provide long-term solid fusion and stability.
- The new cage can achieve good and reliable clinical and radiological outcomes.
- The new cage can be replaced completely by new bone formation.
- The new cage has less complication.

## 1 Introduction

Anterior cervical discectomy and fusion (ACDF) is a gold standard for the treatment of degenerative disc disease associated with radiculopathy or myelopathy.^[1,2]^ The advantages of fusion include: maintaining cervical lordosis, achieving indirect decompression through enlarging the diameter of intervertebral foramen, stabilizing surgical segment, and preventing the progression of posterior lesions.^[3,4]^ In the past decades, autologous tricortical iliac bone graft had always been the preferred bone grafting material. Although this demonstrates high fusion rate, the serious complications of the donor site cannot be ignored.^[5,6]^ In order to avoid these deficiencies, surgeons focus their attention on other graft materials.^[7,8]^ In recent years, various interbody fusion cages of different shapes and materials (titanium, PAMMA, PEEK etc) have been more widely applied in anterior cervical fusion. A lot of clinical research and biomechanical testing exhibited excellent performance and effectiveness; however, for each device, there are inherent deficiencies of raw materials.^[9–11]^

Due to excellent osteoconductive properties and prominent clinical efficacy, allograft bone interbody cages are causing concern. In a prospective semi-random study, the authors compared the allogeneic fibula ring with the autogenous iliac in the ACDF procedure, and found that there was no difference in the fusion rate at the 2-year postoperative follow-up, while fusion time with the allogeneic fibula ring was longer. At the same time, the subsidence also showed no difference. However, the early allograft interbody cage (femoral ring, fibula ring, etc) did not match the anatomical shape of the intervertebral space, had insufficient contact surface with the endplate, easily led to subsidence or extrusion, and pseudarthrosis appeared frequently. In addition, restricted by the properties of raw materials, there were many problems such as irregular specifications, mechanical differential or inappropriate size, which mean that it is difficult to meet the standardized operation of spinal surgery.

In view of the above-mentioned shortcomings, the allograft interbody cage which accords with the anatomical features of intervertebral space is gradually applied to clinic and has achieved favorable results. However, few reports currently involve the cervical anatomic allograft cage. Therefore, we developed a new allograft interbody cage (BioCage), and assessed the clinical and radiological results of the new cage in 1 or 2-level ACDF by comparing with PEEK cage.

## 2 Methods

### 2.1 Study design

Between 2015 and 2016, a prospective non-randomized controlled study of 107 consecutive cases with spondylotic radiculopathy or myelopathy undergoing one to 2-level ACDF using the BioCage or PEEK cage was performed in our institution. Patients had not suffered any cervical surgery before this operation, and those who had etiologies such as fractures, deformity, infections or tumors, severe osteoporosis or the need for a posterior approach, those with chronic systemic illnesses such as diabetes mellitus, rheumatic immune disease, and neurodegenerative diseases, and those with affected segments at more than one level or a follow-up period of less than 12 months were excluded from the research. Patient characteristics are shown in Table 1. Inclusion criteria were radiculopathy, myelopathy, and radiculopathy with myelopathy. Patients diagnosed with other degenerative diseases such as stenosis or arthritis were not ruled out, as long as the diagnosis indicated that the main cause of the complaint was consistent with the clinical nerve root or spinal cord compression.

**Table 1:**
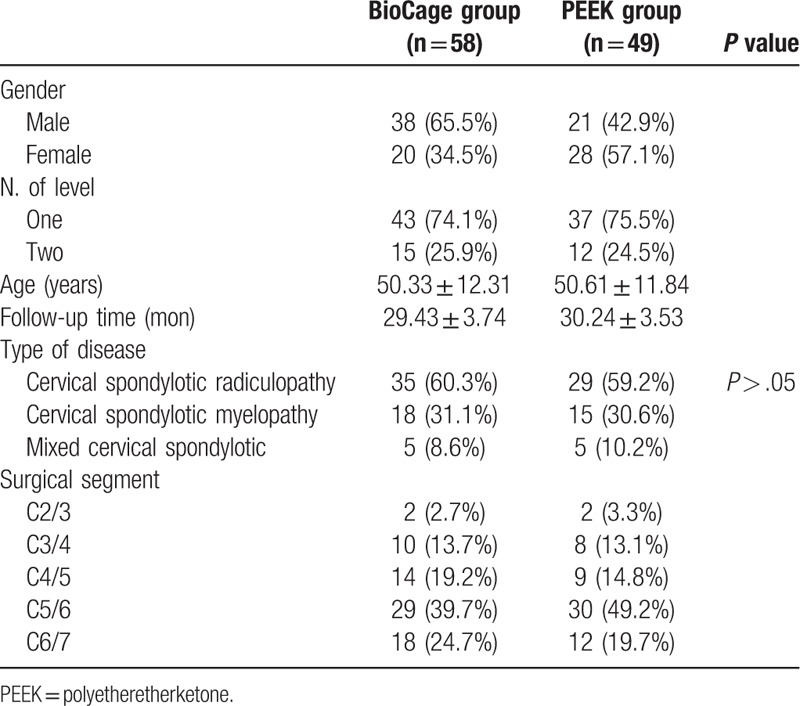
Patient demographics of two groups.

The study was approved by the Ethical Committee of the First Affiliated Hospital, Third Military Medical University (Protocol number: KY201301). All participants signed an informed consent form and all research activities adhered to the tenets of the Declaration of Helsinki.

### 2.2 The BioCage

**Figure 1:**
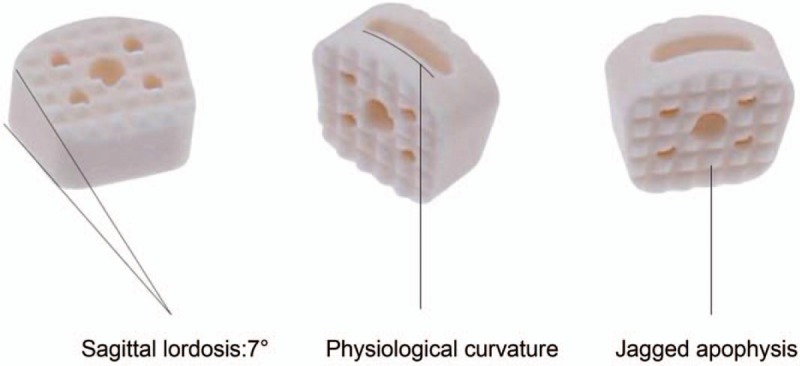
The shape design of BioCage.

The BioCage is a cervical interbody fusion device made of an allograft that was decellularized, degreased, and deep frozen in the bone bank in our hospital. The upper and lower surface of the cage has a certain radians, which makes it more compatible with the anatomical structure of endplate and avoids stress shielding. The utilization rate of raw materials can be greatly improved by using 2 pieces of cortical bone splicing technology, and achieve industrialization (Fig. 1). The static pressure test of ASTM F2077-03 showed that the performance of compressive resistance was much higher than that of the normal endplate. After analysis of the structural mechanical strength by finite element, the optimized bone graft window was designed to facilitate the uniform distribution of stress.

The BioCage has the advantages of biological activity, biomechanics and biocompatibility, and uses osteoconduction and osteoinduction to promote intervertebral fusion, so that requirements for the quality and quantity of the bone graft are low, avoid trauma and complications at the donor site, shorten the operation time, improve the operation efficiency, and can better promote spinal intervertebral fusion.

### 2.3 Surgical procedure

All of the operations were performed by the same team. The surgical procedure was performed using the method described by Smith and Robinson.^[12]^ After radiographic confirmation of the target segment, a completed discectomy and decompression was implemented. Hyperplastic osteophytes were carefully removed for cage filling. The posterior longitudinal ligament was opened in all patients, and the neural structure was decompressed. The intraoperative cage trial was carried out using the templates after distracting the disc space using the Casper system. Then, the cages filled with autogenous bone, which was obtained from decompression, were impacted into the intervertebral space and the appropriate segment was fixed using anterior locked screws and plates. The postoperative processing measures included discharge 24 hours after surgery, preventing infection, nutritional nerve, and rehabilitation therapy. All patients were permitted to stand up the day after surgery and did not require neck collar fixation.

### 2.4 Clinical and radiological evaluation

**Figure 2:**
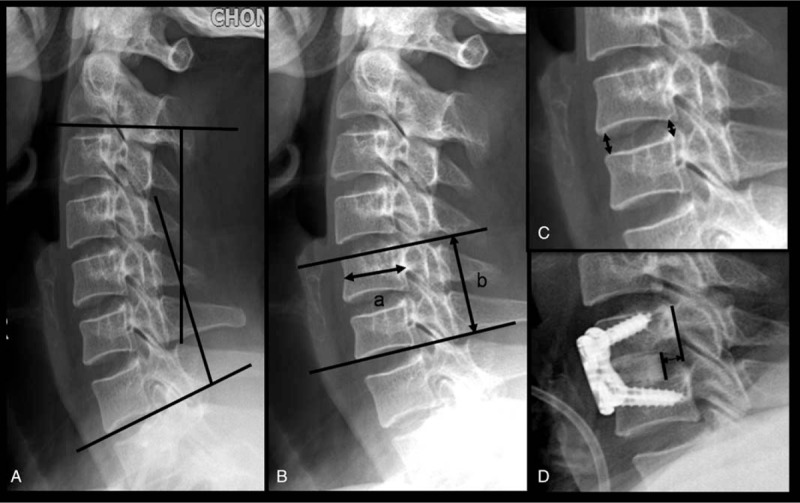
Diagrammatic drawing of measurement data. (A) Overall cervical lordosis: the Cobb angle from the C2 inferior endplate to the C7 inferior endplate. (B) The total vertical height of 2 vertebral bodies divided by the anterior-posterior diameter of the upper vertebral body on a lateral radiograph (IHR = b/a). (C) The anterior and posterior intervertebral height (FSH). (D) The distance between the posterior margin of the cage and the wall of the vertebralbody (D-CAW).

During follow-up, clinical and radiographic results were collected by 1 independent orthopedic surgeon before and after surgery, on the last day of the hospital stay, at postoperative1, 3, 6, and 12 months, and up to the last follow-up. X-ray (antero-posterior and lateral radiographs) and computed tomography (CT) were obtained at postoperative 3, 6, and 12 months to evaluate fusion results (Fig. 2). We define fusion as the presence of bony trabeculation across the fusion level (bony bridging) and a lack of bony lucency at the juncture of the cage and vertebralbody. The absence of such bridges or the presence of an anterior–posterior discontinuation was classified as non-fusion.^[13]^ The VAS and NDI were used to assess the clinical findings preoperatively, postoperatively and at final follow-up. Dysphagia was graded depending on the patient's state as none, mild, moderate, and severe.^[1,14]^

Two independent roentgenologists assessed segmental and overall cervical lordosis, the intervertebral height, IHR, cage positioning, and fusion state. The intervertebral height was measured as the average of the anterior and posterior intervertebral height, as reported by Lee and Gillis.^[15,16]^ Overall cervical lordosis was defined as the Cobb angle from the C2 inferior endplate to the C7 inferior endplate. Segmental cervical lordosis was measured as the facet sagittal angle (FSA) from the upper endplate of superior vertebral body to the lower endplate of the inferior vertebral body of the treated segments. IHR refers to the total vertical height of 2 vertebral bodies divided by the anterior-posterior diameter of the upper vertebral body on a lateral radiograph, and D-PCW is the distance between the posterior margin of the cage and the wall of the vertebralbody (Fig. 2). The data measured by the 2 roentgenologists was averaged for statistical analysis.

### 2.5 Statistical analysis

All analyses were performed using SPSS 19.0. We computed means and SDs for continuous data (VAS, NDI, segmental and overall cervical lordosis (Cobb), and intervertebral height). The index was recorded in preoperative, postoperative and final follow-up. The above indexes were compared with analysis of variances, then using the Dunnett T3 test or LSD *t* test. IHR and D-CAW were analyzed using the Mann–Whitney nonparametric test. Difference between the 2 groups was analyzed by *t* test (α = 0.05)

## 3 Result

**Figure 3:**
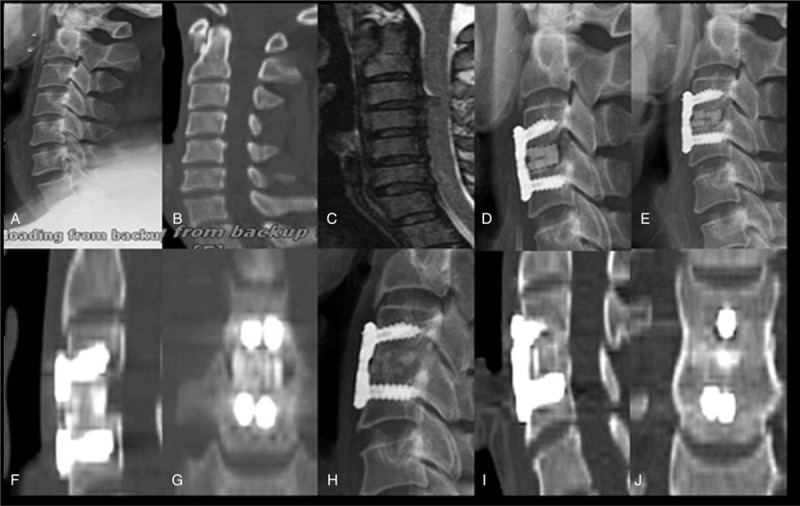
The graph shows a case using BioCage for ACDF. The bone fusion can be achieved at 3 months postoperative. At the last of follow-up, the cage was replaced completely by new bone formation. A 46-year-old man with cervical spondylotic myelopathy. (A–C) preoperative lateral X-ray films, preoperative computed tomography (CT) and magnetic resonance imaging (MRI); (D) lateral X-ray films 3 days after operation; (E-G) X-ray 3 months after operation and CT 3 months after operation; (H-J) X-ray 46 months after operation and CT 46 months after operation.

**Figure 4:**
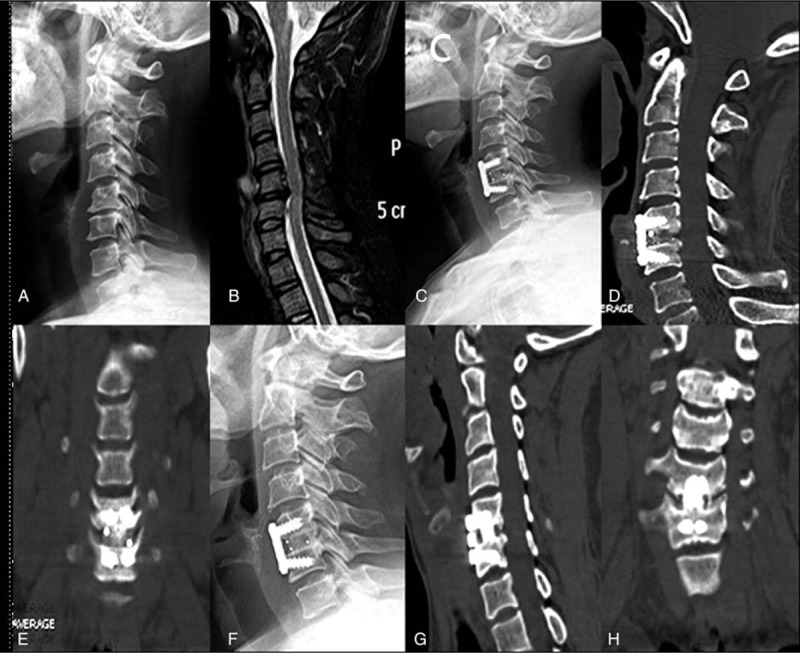
The graph shows a case using PEEK cage for ACDF. There is bony lucency at the juncture of the cage and vertebralbody on CT 3 months after operation, while which disappear at the time of 6 months after operation. A 47-year-old woman with cervical spondylotic myelopathy. (A, B) preoperative lateral X-ray films and preoperative magnetic resonance imaging (MRI); (C-E) X-ray 3 months after operation and CT 3 months after operation; (F-H) X-ray 6 months after operation and CT 6 months after operation.

All patients were followed-up at an average of 29.68 months (range, 12–40 months). VAS and NDI were significantly decreased postoperatively and at the last follow-up in both groups (*P* < .05 compared to pre-op) (Table 2). One patient in group A suffered from cerebrospinal fluid leakage, and was healed by symptomatic treatment. Dysphagia was still emerging in the early postoperative period, 2 and 4 patients (3.45%, 8.16%) complained of minor dysphagia with a duration of 5 to 7 days respectively. Luckily, all patients were resolved by the 12-month follow-up. Postoperative 3 months, group A all achieved bone fusion according to the CT image, the fusion rate was 100% (58/58), 4 cases of group B have not been fusion (1 case of single segment, 3 cases of double segment), the fusion rate was 91.8% (45/49), six month postoperatively, 2 cases in PEEK group have not been fusion (double section), and the fusion rate was 95.9% (47/49). The PEEK group achieved a fusion rate of reached 100% (49/49) at 12 months. Seven cases of allograft group were followed up for more than 3 years, among them we can observe high-density image inside the implanted allograft cages on CT is replaced by continuous cortical bone between the adjacent vertebral endplate. (Figs. 3 and 4).

**Table 2:**
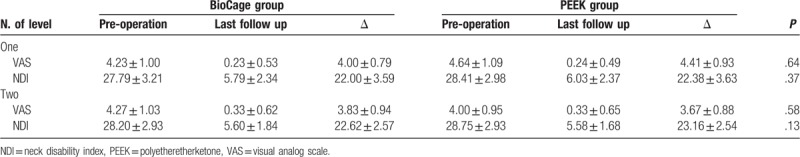
Comparison of clinical outcomes improvement.

The segmental lordosis and overall lordosis were significantly improved immediately after operation; although there was a degree of angle loss at the end of the follow-up, no statistical differences were observed (Table 4). The intervertebral height was significantly improved from 4.52 ± 0.93 mm measured preoperatively to 8.01 ± 0.98 mm immediately after surgery, and decreased to 6.40 ± 0.76 mm at 12 months post-operation in group A, while which was 4.39 ± 0.77 mm, 7.68 ± 0.94 mm, 6.02 ± 0.57 mm, respectively in group B. In the 1-year follow-up, the height presented a declining when radiographs were compared with the immediate postoperative X-rays during the follow-up (*P* < .05). In both groups, the IHR was significantly improved compared with immediate postoperative IHR (*P* < .05), and was also maintained at the final follow-up (*P* < .05) (Table 3). There is no obvious change in D-PCW between immediate postoperative and the 1-year follow-up (*P* > .05). In the research, there were no cage-related complications at the last follow-up.

**Table 4:**
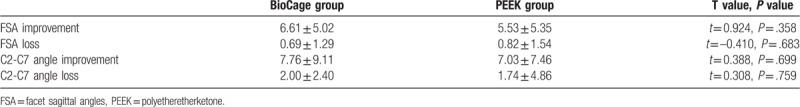
The result of cervical angle.

**Table 3:**
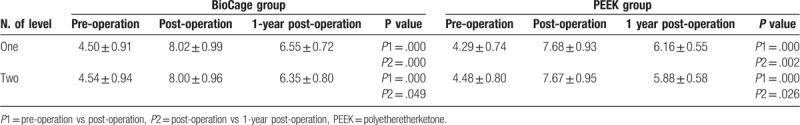
The result of fusion segment height.

## 4 Discussion

Anterior cervical discectomy and fusion is safe and effective for the treatment of degenerative discs of cervical spine with radiculopathy, myelopathy and spondylosis. Although some research has shown that the therapeutic effect was not necessarily associated with the presence of non-fusion, more experimental evidence supported firm fusion as being essential to provide stability for the motion segment and achieve optimal efficacy. Thus, identifying the optimal graft material is indispensable to achieve radiographic fusion.

Conventionally, tricortical autogenous iliac graft is considered the gold standard for fusion. As an interbody fusion material, it can provide osteogenic, osteoinductive and osteoconductive properties to grafts along with mechanical strength, and achieve satisfactory fusion rates. However, the high complication rates at the donor site, and limited sources could be a potential disadvantage.^[17]^ Interbody cages provide immediate stability, high fusion rates and, by filling the intervertebral space, require less structural bone grafts, avoid the harvesting of autogenous bone graft, and may reduce the morbidity associated with the graft-site. Unfortunately, every cage has inherent defects. For example, Titanium cage: the elastic modulus is far greater than normal bone tissue, osteoconduction is poor, and is not conducive to bone formation and fusion. In addition, it is difficult to evaluate the fusion situation owning to non-radiable performance. Furthermore, the stress surface is small, easily leading to the loss of intervertebral height and cage collapse, which may aggravate the symptoms of nerve root compression symptoms.^[18,19]^ Carbon fiber cage: the elastic modulus is closer to normal bone tissue, and stress shielding is smaller. However, the detached tissue structure may cause inflammatory reaction, which affects the postoperative healing, and is fragile and delicate.^[20]^ Bioabsorbable cage: it has small stress shielding, radiability, and absorbability, but has some defects including chronic inflammatory reaction, material absorption and unsynchronized osteogenesis. Polyetheretherketone (PEEK) cage: it possesses high mechanical strength and radiability, simultaneously, elastic modulus is similar to cortical bone, friction performance is excellent, and biocompatibility is good. Thus, the PEEK cage is the most widely used intervertebral implant in ACDF at present.^[21,22]^ However, it is undeniable that as a kind of polymeric material, PEEK does not have the three-dimensional mesh structure for osteogenesis, which is adverse to the ostealcreeping substitution. In addition, as a foreign matter, it exists in the intervertebral space, occupying the space for the fusion of normal bone tissue. Theoretically, it will inevitably affect the fusion firmness. Therefore, the PEEK material is not perfect for the production of fusion cages.

Due to deficiencies in the materials described above, there is a need for an interbody cage with a strength that is close to that of bone, with good osteoconduction and osteoinductivity. Therefore, increasing attention has been paid to allograft bone interbody fusion. At present, the allogeneic bone used in clinic is mainly cortical bone, with a natural 3-dimensional mesh structure, containing various growth factors required for osteogenesis. While preserving good osteogenic capacity, it can also provide axial support of the anterior column and avoid intervertebral space descent. In recent years, allogeneic bone interbody cages, which show similarities with the anatomical characteristics of the intervertebral space, have been gradually used in interbody fusion surgery, avoiding defects in the shape and performance of traditional allografts, and have obtained remarkable efficacy. Arnold^[23]^ reported an allograft cage for posterior fusion in lumbar degenerative disease, which is made of two pieces of cortical bone and a tooth-shaped surface. In a subsequent multicenter prospective study, Arnold found that 98% of patients achieved fusion 1 year postoperatively. Kao et al^[24]^ assessed 73 patients using allografts, autologous iliac crest grafts, or cages in cervical discectomies and interbody fusions. The results showed that the fusion rates of allografts were up to 97.6%. In the present research, we used the BioCage to treat 58 patients with cervical spondylosis (73 segments), with a fusion rat of 100% at 3 months postoperatively, while the fusion rate of PEEK cage was 91.8%, showing better fusion capability.

Subsidence is an important problem that should be focused on. Barsa et al^[25]^ found that 13.2% of patients had significant subsidence after ACDF, while Bartel^[26]^ reported a higher subsidence rate (29.2%). Our results confirmed that an acceptable degree of cage subsidence happened at the 1-year follow-up of all patients. The average subsidence of BioCage was 1.71 mm. Mean subsidence of PEEK cage was 1.66 mm. There was no statistical difference between them. Previous literature reported that there was no significant correlation between the clinical results and the subsidence of the cage.^[26,27]^ In our research, none of the patients with cage subsidence suffered any associated clinical symptoms and this also did not have a significant impact on bone fusion.

Another important factor affecting the postoperative clinical outcome is cervical sagittal balance. The cervical sagittal balance is very important to maintain the global sagittal balance, and the loss of physiological curvature will lead to pain and dysfunction, which may increase the risk of adjacent segment degeneration.^[3,28]^ Gillis et al retrospectively analyzed 74 cases with 1-level or 2-level ACDF using an allograft combined plate. They found a mean change in C2–7 lordosis of 2.34° postoperatively and 3.46° at the 1-year follow-up; segmental lordosis gave a mean improvement of 6.31° at postoperative 6 weeks and 6.45° at 1 year. Kulkarni et al^[2]^ reviewed 15 consecutive cases of single-level anterior cervical interbody fusion using the PEEK cage for cervical spondylotic radiculopathy or myelopathy. The immediate postoperative lordotic angle was greater than the preoperative angle; at the last follow-up, the angle was less than the value at the immediate postoperative period, and the average improved angle was only 0.9° compared with the preoperative angle. In our research, segmental and overall cervical lordosis were both significantly greater immediately post-operation than pre-operation. Although the segmental lordosis and overall lordosis had decreased at the last follow-up, this was not statistically significant.

In the present study, BioCage has achieved satisfactory clinical results, a high fusion rate and fewer complications; however, we also acknowledge the limitations of the research. First the sample size was small and the follow-up was short. Second, muti-level cases were not involved, making the research less convincing. More, especially long-term follow-up cases are needed to provide more valuable information.

## 5 Conclusion

The fusion rate of BioCage is comparable to the previous researches of autologous iliac crest grafts and better than PEEK cages. No significant difference is observed in the cervical lordosis, intervertebral height and rate of subsidence between the groups. Although the BioCage has shown excellent efficacy in clinical application, there is still a lack of long-term follow-up to judge the further effect. The pathological changes and radiographic findings of allografts in the fusion process are different from the metal or synthetic material cages, so we still need to further study the optimal indications and long-term efficacy, and provide sufficient evidence for clinical decision-making.

## Acknowledgments

The authors thank the hospital fund management of the First Affiliated Hospital of the Third Military Medical University.

## Author contributions

**Conceptualization:** Yang Yu.

**Data curation:** Sen Yang, Xun Liu, Wenjie Wu.

**Formal analysis:** Yang Yu, Xun Liu, Wenjie Wu.

**Funding acquisition:** Jianzhong Xu.

**Investigation:** Sen Yang, Zehua Zhang, TianYong Hou, Jianzhong Xu, Wenjie Wu.

**Methodology:** Sen Yang, Wenjie Wu.

**Project administration:** Sen Yang, Zehua Zhang, TianYong Hou, Jianzhong Xu, Wenjie Wu.

**Resources:** Sen Yang, Xun Liu.

**Supervision:** Jianzhong Xu.

**Writing – original draft:** Sen Yang, Yang Yu, Wenjie Wu.

**Writing – review & editing:** Fei Luo.
